# Deciphering the dynamic single-cell transcriptional landscape in the ocular surface ectoderm differentiation system

**DOI:** 10.1093/lifemedi/lnae033

**Published:** 2024-09-05

**Authors:** Canwei Zhang, Zesong Lin, Yankun Yu, Siqi Wu, Huaxing Huang, Ying Huang, Jiafeng Liu, Kunlun Mo, Jieying Tan, Zhuo Han, Mingsen Li, Wei Zhao, Hong Ouyang, Xiangjun Chen, Li Wang

**Affiliations:** State Key Laboratory of Ophthalmology, Zhongshan Ophthalmic Center, Sun Yat-sen University, Guangdong Provincial Key Laboratory of Ophthalmology Visual Science, Guangzhou 510060, China; Department of Ophthalmology, The First Affiliated Hospital of Shandong First Medical University & Shandong Provincial Qianfoshan Hospital, Jinan 250014, China; State Key Laboratory of Ophthalmology, Zhongshan Ophthalmic Center, Sun Yat-sen University, Guangdong Provincial Key Laboratory of Ophthalmology Visual Science, Guangzhou 510060, China; State Key Laboratory of Ophthalmology, Zhongshan Ophthalmic Center, Sun Yat-sen University, Guangdong Provincial Key Laboratory of Ophthalmology Visual Science, Guangzhou 510060, China; Department of Pathology, The First Affiliated Hospital of Shihezi University, Shihezi 832002, China; State Key Laboratory of Ophthalmology, Zhongshan Ophthalmic Center, Sun Yat-sen University, Guangdong Provincial Key Laboratory of Ophthalmology Visual Science, Guangzhou 510060, China; State Key Laboratory of Ophthalmology, Zhongshan Ophthalmic Center, Sun Yat-sen University, Guangdong Provincial Key Laboratory of Ophthalmology Visual Science, Guangzhou 510060, China; State Key Laboratory of Ophthalmology, Zhongshan Ophthalmic Center, Sun Yat-sen University, Guangdong Provincial Key Laboratory of Ophthalmology Visual Science, Guangzhou 510060, China; State Key Laboratory of Ophthalmology, Zhongshan Ophthalmic Center, Sun Yat-sen University, Guangdong Provincial Key Laboratory of Ophthalmology Visual Science, Guangzhou 510060, China; State Key Laboratory of Ophthalmology, Zhongshan Ophthalmic Center, Sun Yat-sen University, Guangdong Provincial Key Laboratory of Ophthalmology Visual Science, Guangzhou 510060, China; State Key Laboratory of Ophthalmology, Zhongshan Ophthalmic Center, Sun Yat-sen University, Guangdong Provincial Key Laboratory of Ophthalmology Visual Science, Guangzhou 510060, China; State Key Laboratory of Ophthalmology, Zhongshan Ophthalmic Center, Sun Yat-sen University, Guangdong Provincial Key Laboratory of Ophthalmology Visual Science, Guangzhou 510060, China; State Key Laboratory of Ophthalmology, Zhongshan Ophthalmic Center, Sun Yat-sen University, Guangdong Provincial Key Laboratory of Ophthalmology Visual Science, Guangzhou 510060, China; Center for Stem Cell Biology and Tissue Engineering, Key Laboratory for Stem Cells and Tissue Engineering, Ministry of Education, Zhongshan School of Medicine, Sun Yat-Sen University, Guangzhou 510080, China; State Key Laboratory of Ophthalmology, Zhongshan Ophthalmic Center, Sun Yat-sen University, Guangdong Provincial Key Laboratory of Ophthalmology Visual Science, Guangzhou 510060, China; Center for Stem Cell Biology and Tissue Engineering, Key Laboratory for Stem Cells and Tissue Engineering, Ministry of Education, Zhongshan School of Medicine, Sun Yat-Sen University, Guangzhou 510080, China; Eye Center of the Second Affiliated Hospital, Zhejiang University School of Medicine, Hangzhou 310009, China; Institute of Translational Medicine, Zhejiang University School of Medicine, Hangzhou 310020, China; State Key Laboratory of Ophthalmology, Zhongshan Ophthalmic Center, Sun Yat-sen University, Guangdong Provincial Key Laboratory of Ophthalmology Visual Science, Guangzhou 510060, China

**Keywords:** ocular surface ectoderm, embryonic stem cells, surface ectoderm, single-cell transcriptomics

## Abstract

The ocular surface ectoderm (OSE) is essential for the development of the ocular surface, yet the molecular mechanisms driving its differentiation are not fully understood. In this study, we used single-cell transcriptomic analysis to explore the dynamic cellular trajectories and regulatory networks during the *in vitro* differentiation of embryonic stem cells (ESCs) into the OSE lineage. We identified nine distinct cell subpopulations undergoing differentiation along three main developmental branches: neural crest, neuroectodermal, and surface ectodermal lineages. Key marker gene expression, transcription factor activity, and signaling pathway insights revealed stepwise transitions from undifferentiated ESCs to fate-specified cell types, including a PAX6 + TP63 + population indicative of OSE precursors. Comparative analysis with mouse embryonic development confirmed the model’s accuracy in mimicking *in vivo* epiblast-to-surface ectoderm dynamics. By integrating temporal dynamics of transcription factor activation and cell–cell communication, we constructed a comprehensive molecular atlas of the differentiation pathway from ESCs to distinct ectodermal lineages. This study provides new insights into the cellular heterogeneity and regulatory mechanisms of OSE development, aiding the understanding of ocular surface biology and the design of cell-based therapies for ocular surface disorders.

## Introduction

The ocular surface ectoderm (OSE) is the source of the ocular surface epithelium, including the corneal epithelium, conjunctival epithelium, and limbal stem cells [1, 2]. These cells are crucial for maintaining vision and overall ocular function by ensuring the integrity of the cornea and conjunctiva, tear film stability, and protection against environmental insults [3–5]. Disruptions in the normal development of the OSE can lead to various inherited anterior segment eye diseases, highlighting the importance of understanding the mechanisms underlying its development and maintenance [6–11].

ESCs are characterized by their pluripotency and their ability to differentiate into all three germ layers: ectoderm, mesoderm, and endoderm [12–14]. The differentiation potential of ESCs into ectodermal lineages, which includes the neural crest, neuroectoderm, and surface ectoderm, makes them an invaluable model for studying early developmental processes of eye tissue [12, 15]. The differentiation process of ESCs into ectodermal lineages is orchestrated by a complex interplay of signaling pathways and transcription factors [13, 16, 17]. Key molecules such as PAX6, TP63, and signaling pathways including Wnt, BMP, and Notch have been identified as critical regulators of this process [12, 18–21]. However, despite these advances, the precise molecular mechanisms and cellular dynamics that govern OSE differentiation remain incompletely understood [22].

Many studies have traditionally relied on bulk RNA sequencing and other methods that average out cellular heterogeneity, potentially masking critical insights into the differentiation process [23]. Single-cell RNA sequencing (scRNA-seq) has revolutionized the field by allowing researchers to dissect tissues at a single-cell resolution, thereby uncovering the diversity of cell states and the transitions between them [24]. This technology has been successfully applied to map the differentiation trajectories of various stem cell systems, providing an elaborate view of cellular differentiation and lineage specification [25–28]. For instance, a comprehensive study of mouse gastrulation and early organogenesis utilized scRNA-seq to profile 116,312 single cells, constructing a molecular map of cellular differentiation from pluripotency toward all major embryonic lineages, and highlighting the utility of this method in understanding developmental processes [29].

Understanding the single-cell transcriptional landscape of OSE differentiation is crucial for advancing our knowledge of ocular surface biology. This understanding can inform the development of targeted therapies for ocular surface disorders, which currently lack effective treatments [5, 30]. In this study, we utilized scRNA-seq to delineate the dynamic cellular trajectories and regulatory networks during the *in vitro* differentiation of ESCs into the OSE lineage. Through unbiased clustering and trajectory inference, we identified distinct cell subpopulations and developmental branches, providing a detailed map of the differentiation process. By integrating the temporal dynamics of transcription factor activation and cell–cell communication signals, we constructed a comprehensive molecular atlas tracing the differentiation pathway from ESCs to distinct ectodermal lineages. This study fills significant knowledge gaps by offering unprecedented insights into the cellular heterogeneity, lineage trajectories, and key regulatory mechanisms governing OSE development.

## Results

### Establishment of the differentiation system from ESC to OSE

To decipher the dynamic molecular characteristics of the OSE commitment, we developed a robust protocol to differentiate human embryonic stem ESCs into ectodermal lineages and characterized this process using phase-contrast microscopy, and immunofluorescence staining. The human ESC cell line H1 (WA-01) was cultured on Matrigel-coated plates using mTeSRTM1 medium. Differentiation of ESCs began with dissociation using gentle cell dissociation reagent (GCDR), followed by seeding cell aggregates on LN511E-coated plates and culturing in mTeSRTM1 medium for 3–7 days. The medium was then switched to differentiation medium (DM) and cells were cultured for 3 weeks, yielding OSE, and general surface ectoderm (GSE) cells. For further differentiation into corneal epithelial cells, the cells were cultured in corneal differentiation medium (CDM) for 6 weeks, followed by an additional 2 weeks in corneal epithelium culture medium (CECM) (Fig. S1A). A self-formed ectodermal autonomous multi-zone (SEAM) was generated as reported in the induced pluripotent stem cells (iPSCs) differentiation induction system [3, 31]. Sequential phase-contrast microscopy images captured the morphological changes of ESCs throughout the early stages of differentiation, spanning from Day 0 to Day 11 (Fig. S1B).

**Figure 1.**
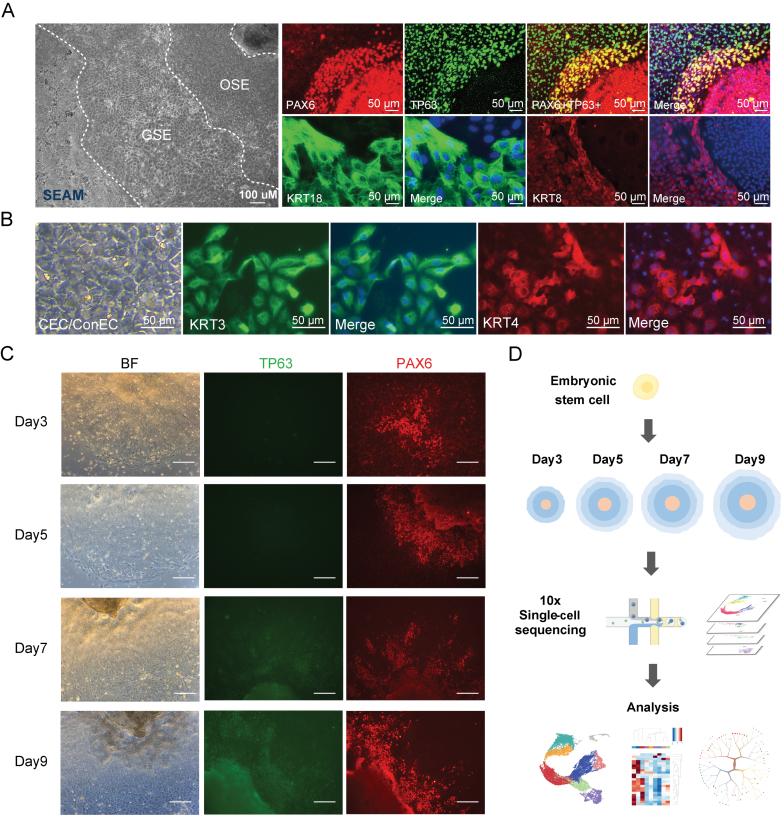
**Establishment of the differentiation system from ESC to OSE and the dynamic single-cell sequencing strategy.**(A) Phase-contrast image showing the differentiation of ESC into multiple zones of OSE and GSE in SEAM morphology along with immunofluorescence staining for PAX6, TP63, KRT8, and KRT18. (B) Bright-field image of further differentiated OSE cells into CEC or ConEC with immunofluorescence staining for KRT3 and KRT4. (C) Time-course immunofluorescence images showing the expression of key transcription factors TP63 and PAX6 at four early time points (Days 3, 5, 7, and 9) during induced differentiation into OSE cells. (D) Schematic diagram of the single-cell RNA sequencing approach used to analyze the dynamic differentiation pathway from human embryonic stem cells over time (Days 3, 5, 7, and 9). Scale bars: 50 μm.

We monitored the differentiation of ESCs into multiple epithelial zones through phase-contrast microscopy. In the resulting SEAM (surface epithelium and ectodermal morphology) structure, distinct regions of OSE and general GSE were observed (Fig. 1A). Immunofluorescence staining was used to further validate the differentiation process by detecting the expression of key epithelial markers. PAX6 and TP63, crucial markers of the ocular surface epithelial lineage, were abundantly present (Fig. 1A), indicating the presence of epithelial progenitor cells. The presence of KRT8 and KRT18 cytokeratins confirmed the surface ectoderm identity of these cells (Figs. 1A and S1C), indicating successful differentiation into OSE and GSE cells. As the differentiation progressed, the OSE cells further differentiated into more specialized ocular epithelial cells, including corneal epithelial cells (CEC) and conjunctival epithelial cells (ConEC). Bright-field microscopy revealed significant morphological different characteristics of these cell types (Fig. 1B). Subsequent immunofluorescence analysis confirmed their identity at the molecular level, revealing the expression of KRT3, a specific marker for corneal epithelial cells, and KRT4, a marker for conjunctival epithelial cells (Fig. 1B).

To gain insights into the dynamics of the differentiation process, we conducted a time-course immunofluorescence analysis over four early time points: Day 3, Day 5, Day 7, and Day 9. Bright-field microscopy provided an overview of cell morphology at each stage (Fig. 1C). Immunofluorescence analysis indicated the expression patterns of key transcription factors involved in OSE-differentiation. TP63 expression was mostly noted from Day 7 onwards, indicating its role in the late stages of epithelial progenitor differentiation. Conversely, PAX6 exhibited an early onset from Day 3, with increased intensity over time, reflecting its vital role in ocular surface epithelial lineage commitment (Fig. 1C). These temporal analyses underscore the critical periods of transcription factor activity and identify pivotal regulatory events during OSE differentiation. Overall, we have successfully established a comprehensive ESC to OSE differentiation system.

Further elucidation of the molecular diversification during differentiation was achieved by employing single-cell RNA sequencing (Fig. 1D). A schematic diagram encapsulated the differentiation workflow, with human ESCs undergoing differentiation. Four time points (Day 3, Day 5, Day 7, and Day 9) of differentiation underwent 10x single-cell RNA sequencing followed by rigorous bioinformatics analysis to uncover key regulatory networks and transcriptional changes.

### Single-cell transcriptomic dynamics reveal cell populations and developmental progression in the OSE differentiation system

**Figure 2.**
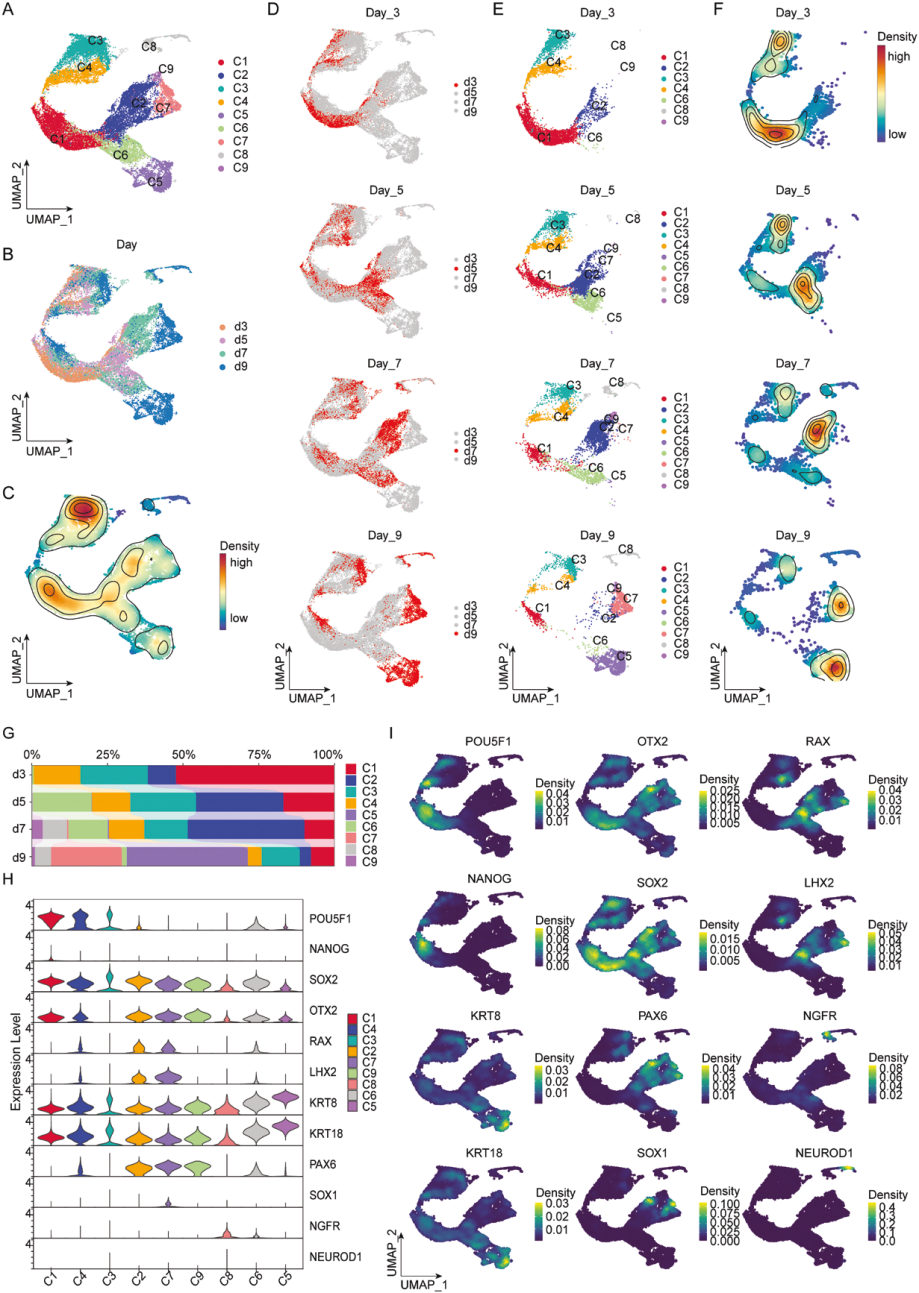
**Single-cell transcriptomic dynamics and cell population identification in OSE differentiation.**(A) UMAP plot showing the nine identified cell subgroups (C1–C9) from the single-cell transcriptome data of the OSE differentiation system. (B) UMAP plot colored by different time points (Day 3, Day 5, Day 7, and Day 9). (C) Spatial density plot of the cell distributions at each time point. (D–F) Time-series facet plots showing the distribution and density of identified single-cell subgroups over time. (G) Bar plot illustrating the proportions of each cell subgroup (C1–C9) at different time points. (H) Violin plot displaying the expression distribution of known ESC markers (POU5F1 and NANOG), highly conserved eye field transcription factors (EFTFs) (OTX2, SOX2, RAX, LHX2, PAX6, and SOX1), surface ectoderm markers (KRT8 and KRT18), and neural crest markers (NGFR and NEUROD1). (I) Density plot depicting the expression levels of these genetic markers on UMAP.

To reveal the single-cell dynamic transcriptional information of the OSE differentiation system, we collected single-cell transcriptome data from the OSE differentiation system at four time points: Day 3, Day 5, Day 7, and Day 9, obtaining a total of 22,328 cells. First, we performed data embedding, uniform manifold approximation and projection (UMAP) dimensionality reduction, and Louvain unsupervised clustering. This unbiased analysis identified nine cell subgroups, labeled C1–C9 (Fig. 2A). Subsequently, the UMAP distributions of different days and spatial densities were displayed (Fig. 2B and 2C). Through the time-series facet plot, we observed that, over time, the distribution of the identified single-cell subgroups gradually dispersed, and the high-density cell areas also became more spread out (Fig. 2D–F). The statistical analysis of the cell subgroup proportions showed that, over time, the proportions of subgroups C1, C3, and C4 gradually decreased, while the proportions of subgroups C5, C7, and C8 gradually increased. The proportions of subgroups C2, C6, and C9 first increased and then decreased (Figs. 2G and S2A). These density distribution and cell proportion results suggest that subgroups C1, C3, and C4 represent undifferentiated stem cell/progenitor cell subgroups, C5, C7, and C8 represent differentiated cell subgroups, and C2, C6, and C9 represent intermediate differentiating cells. Based on the automatic cell annotation results from singleR, C1, C3, C4, and C6 are more closely related to ESCs, while the remaining cell subpopulations are more similar to differentiated neuroepithelial cells or neurons (Fig. S2B), which is consistent with our assessment of the cell differentiation state. The subpopulation and temporal distribution results from the too many cells branching analysis also show that differentiated cells appear and increase over time (Fig. S2C and S2D).

To further explored the differentiation status of the culture system, the expression distribution of known genetic markers of ESCs (POU5F1 and NANOG), highly conserved eye field transcription factors (EFTFs) (OTX2, SOX2, RAX, LHX2, PAX6, and SOX1) [32–36], surface ectoderm markers [16] (KRT8 and KRT18), and neural crest markers [37–39] (NGFR and NEUROD1) were demonstrated (Fig. 2H and 2I). The results showed that ESC markers were highly expressed in C1, EFTFs that related to retinogenesis (OTX2, RAX, LHX2, PAX6, and SOX1) were highly expressed in C2, C7, and C9, surface ectoderm markers that related to ocular surface ectoderm development (KRT8 and KRT18) were highly expressed in C5 and C6, and neural crest markers (NGFR and NEUROD1) were highly expressed in C8. To locate ocular surface ectodermal cells or precursor cells in the culture system, we accessed the UMAP expression of different ectodermal markers through the time-series facet plot. The results revealed that the key ocular surface ectoderm transcription factors TP63 and PAX6 primarily co-appeared on Day 7 and Day 9, distributed within the surface ectodermal populations with high expression of KRT8 and KRT18 (Fig. S3). This is consistent with the established knowledge that the ocular surface ectoderm originates from the surface ectoderm [2, 3].

**Figure 3.**
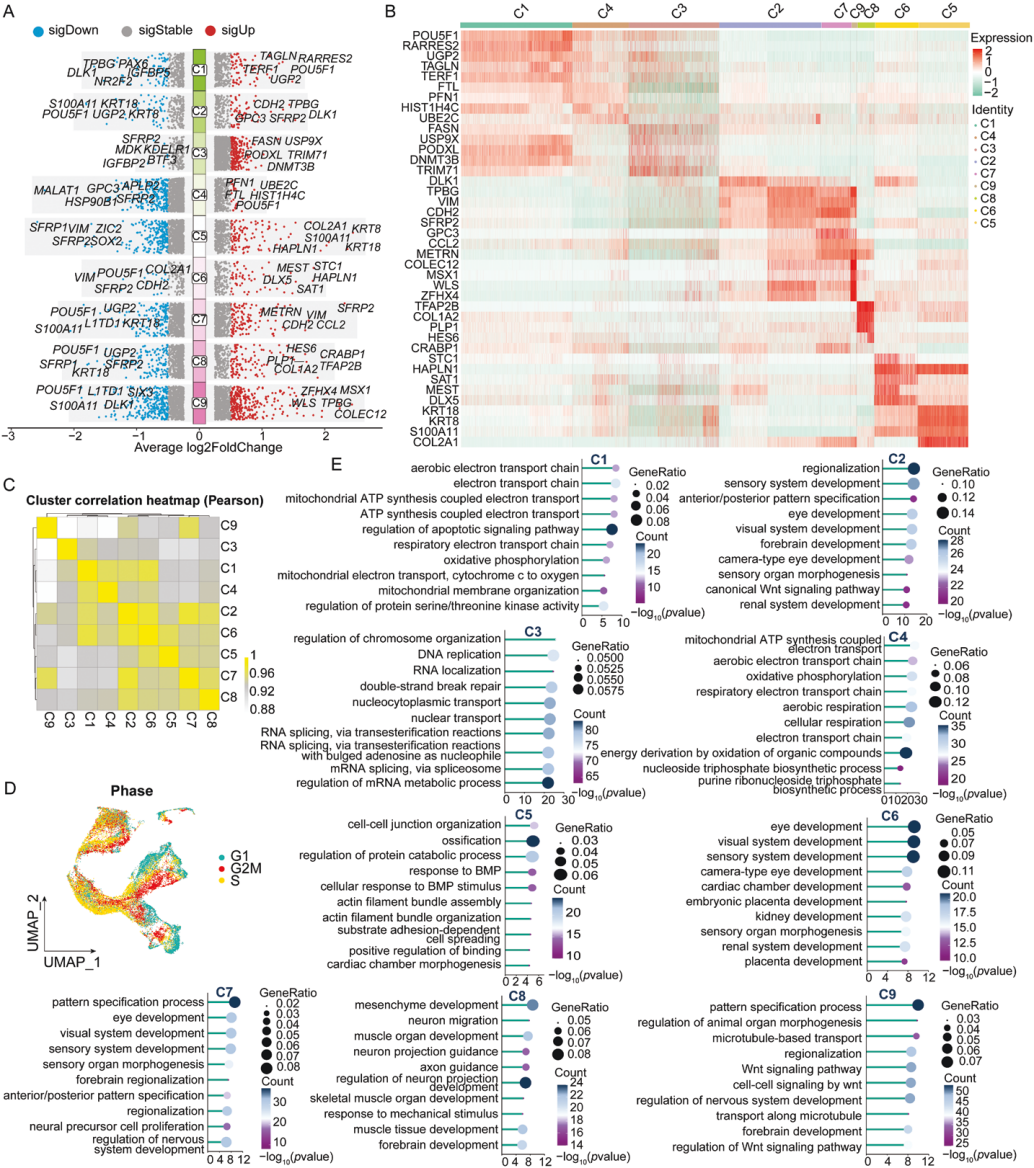
**Differential gene expression and functional characterization of OSE differentiation subgroups.**(A) Volcano plots displaying the top five upregulated and downregulated differential genes for each identified cell subgroup (C1–C9). Significant upregulated genes are labelled in right side, while significant downregulated genes are labelled in left side. (B) Heatmap illustrating the expression differences of the top five differential genes across all cell subgroups, highlighting distinct gene expression patterns among the groups. (C) Pearson correlation heatmap demonstrating the similarity in gene expression patterns among the different cell subgroups. (D) UMAP plots showing cell cycle phase distribution. (E) GO: BP enrichment analysis of upregulated differential genes in each subgroup.

We further investigated the differential genes and characteristics of these unsupervised subgroups. Volcano plots were used to display the top five upregulated and downregulated differential genes in each subgroup, and heatmaps were used to show the expression differences of the top five differential genes in all groups (Fig. 3A and 3B). The results showed that C1, C3, and C4 had similar embryonic stem cell expression patterns, C2, C7, and C9 had similar expression patterns, and C5 and C6 had similar gene expression patterns. The top five differentially expressed genes for each cell subgroup at each time point are displayed in volcano plots in Fig. S4A. Pearson correlation results demonstrated similar patterns (Fig. 3C). UMAP results of cell cycle scoring also indicated that differentiated cell populations tended to be in the G1 phase, while stem/progenitor and intermediate cell populations were more inclined to be in the proliferative G2M and S phases (Fig. 3D). Gene Ontology (GO): Biological Process (BP) enrichment analysis of the upregulated differential genes in all subgroups revealed that the differential genes in stem cell-like C1 and C4 primarily highlighted mitochondrial functions and processes associated with the electron transport chain. The GO terms in C3 were largely involved in nucleic acid metabolism and chromosomal processes, including regulation of chromosome organization, DNA replication, RNA splicing, and processing. In C2, the enriched GO terms reflected a focus on developmental processes and neural differentiation, such as sensory system development, anterior/posterior pattern specification, and visual system development. The clustering of sensory and neural development terms underscored the involvement of genes in organogenesis and tissue patterning. Enrichment in C5 indicates processes involving cell structural organization and BMP signaling pathways. C6 highlights organ developmental processes primarily for sensory and cardiac systems including the eye. C8 enrichment identifies processes related to the development of the mesenchyme and neuron. In both C7 and C9, the GO terms are enriched for processes of pattern specification process, while C7 enrichment includes several processes related to eye development and nervous system development, suggesting a significant role in retinogenesis and patterning during development (Fig. 3E). The Kyoto encyclopedia of genes and genomes (KEGG) pathway analysis results for the top differentially expressed genes of each cell subgroup are presented in Fig. S4B.

### Developmental lineages, signaling pathways, and transcription factors analysis in the OSE differentiation system

**Figure 4.**
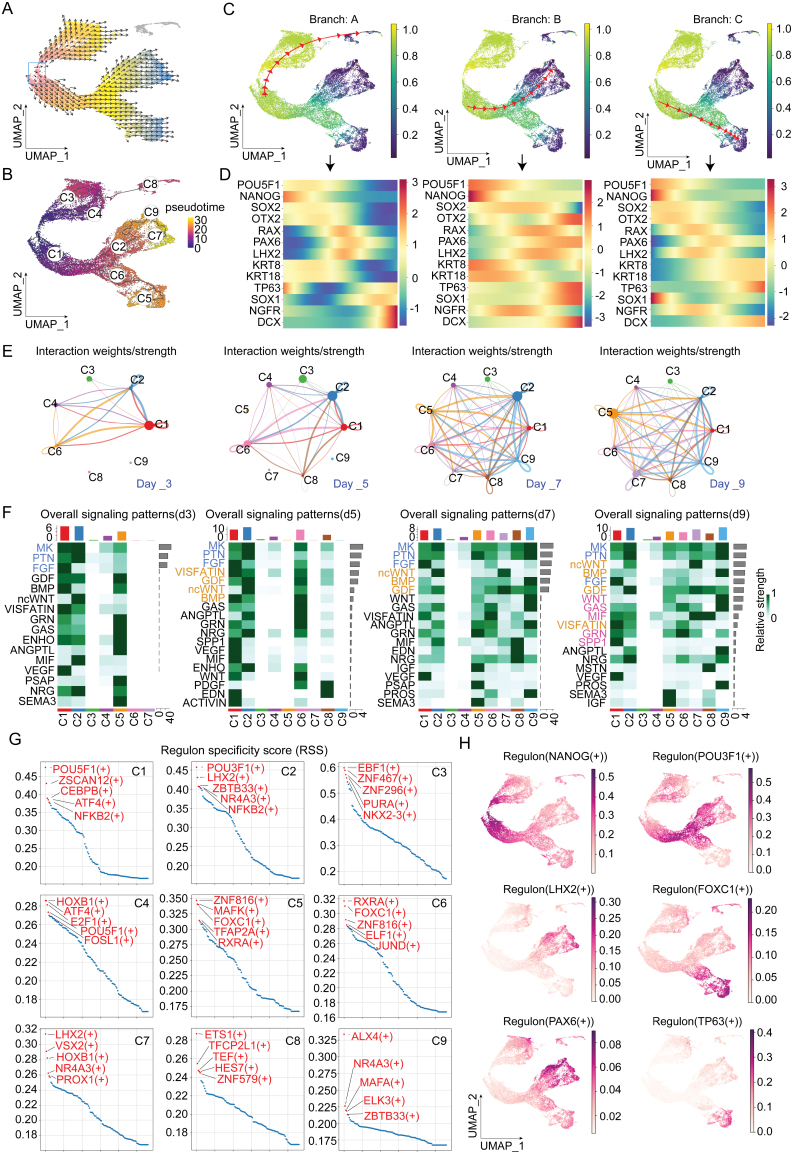
**Developmental lineages, signaling pathways, and transcription factor analysis in the OSE differentiation system.** (A) UMAP plot showing the inferred developmental vectors of cell differentiation within the OSE system using the VECTOR tool. Box showing the initial developmental positions. Arrows indicate the direction of differentiation. (B) Pseudotemporal trajectory analysis using Monocle, illustrating distinct differentiation pathways. Cells are colored according to their pseudotime. (C) Pseudotemporal trajectory analysis using Palantir, showing consistent differentiation pathways identified in Monocle analysis. (D) Heatmap showing the expression changes of key marker genes (POU5F1, NANOG, SOX2, OTX2, RAX, PAX6, LHX2, KRT8, KRT18, TP63, SOX1, NGFR, and DCX) along the three identified differentiation branches. (E) Circle plot showing the total interaction weight/strength between cell subpopulations from Day 3 to Day 9. (F) Heatmap illustrating the dynamic changes in ligand–receptor signaling pathways between cell subpopulations over time by Cellchat. (G) The RSS ranking for key transcription factors in each cell subpopulation was calculated using the pySCENIC algorithm. (H) UMAP plots displaying the transcriptional activity distribution of core transcription factors (NANOG, POU3F1, LHX2, FOXC1, PAX6, and TP63).

Several methods have been employed to identify developmental lineage directions and pseudotemporal pathways within the OSE differentiation system. By utilizing VECTOR, a sophisticated tool designed to infer vectors of developmental directions for cells within UMAP projections [40], we discovered that the initial positions of development are located in cell clusters C1 and C4. The inferred developmental trajectories point towards cell clusters C3, C8, C7, and C5 (Fig. 4A). Additionally, through pseudotemporal inference using advanced algorithms such as Monocle [26, 41] and Palantir [27], we identified three distinct differentiation pathways within the OSE system: Branch A, which follows the trajectory from C1 to C4 to C3 to C8; Branch B, which follows the trajectory from C1 to C2 to C9 to C7; and Branch C, which follows the trajectory from C1 to C6 to C5 (Fig. 4B and 4C). By combining the expression changes of important marker genes (POU5F1, NANOG, SOX2, OTX2, RAX, PAX6, LHX2, KRT8, KRT18, TP63, SOX1, NGFR, and DCX) along these branches (Fig. 4D) and the UMAP expression patterns (Fig. 2I), we identified three main differentiation pathways: Branch A as the neural crest developmental lineage, Branch B as the neuroectodermal developmental lineage, and Branch C as the surface ectodermal lineage. These findings provide a comprehensive understanding of the dynamic transcriptional landscapes and developmental trajectories within the OSE differentiation system.

We further utilized the Cellchat [42] algorithm to explore the changes in ligand–receptor signaling pathways between cell subpopulations over time. Firstly, from day 3 to day 9, the overall interaction weight/strength between cell subpopulations significantly increased (Fig. 4E), and the number of subpopulations with active incoming and outgoing communications also expanded from C1 and C3 on day 3 to most subpopulations by day 9 (Fig. S5A). On day 3, the MK, PTN, and FGF pathways exhibited strong intercellular communication; by day 5, the VISFATIN, GDF, ncWNT, and BMP pathways gradually strengthened; the communication on Day 7 was similar to that on Day 5; and by Day 9, more pathways showed increased activity, including WNT, GAS, MIF, GRN, and SPP1(Fig. 4F). These results suggest that there is a dynamic and progressive enhancement in intercellular communication over time, reflecting complex regulatory mechanisms at play as the cells undergo differentiation and development. The detailed relationships of these active pathways between subpopulations from Day 3 to Day 9 are illustrated in Fig. S5B.

Transcription factor activation is a crucial molecular event in development and differentiation. To identify key transcription factors in the OSE differentiation system, we used the pySCENIC algorithm to calculate the transcription factor scores for each cell subpopulation. As shown in Fig. 4G, the regulon-specific score (RSS) ranking provides insights into the key transcription factors for each cell subpopulation, including both known and newly discovered factors. FOXC1 and RXRA are known to be key transcription factors for the surface ectoderm (C5 and C6), but the relationship of ZNF816, which also has a high RSS score, with surface ectoderm remains unknown. By analyzing the transcriptional activity distribution of several core transcription factors (NANOG, POU3F1, LHX2, FOXC1, PAX6, and TP63) on UMAP, we further confirmed the three differentiation pathways of OSE and the identity of differentiated cells (Fig. 4H). The results of the transcription factor analysis indicate that the differentiation process within the OSE system is regulated by a complex network of both known and novel transcription factors, which orchestrate the progression and specialization of cells along the identified developmental pathways. These transcription factor results warrant further investigation and validation.

### Identification and characterization of neuroectoderm, surface ectoderm, and OSE differentiation

**Figure 5.**
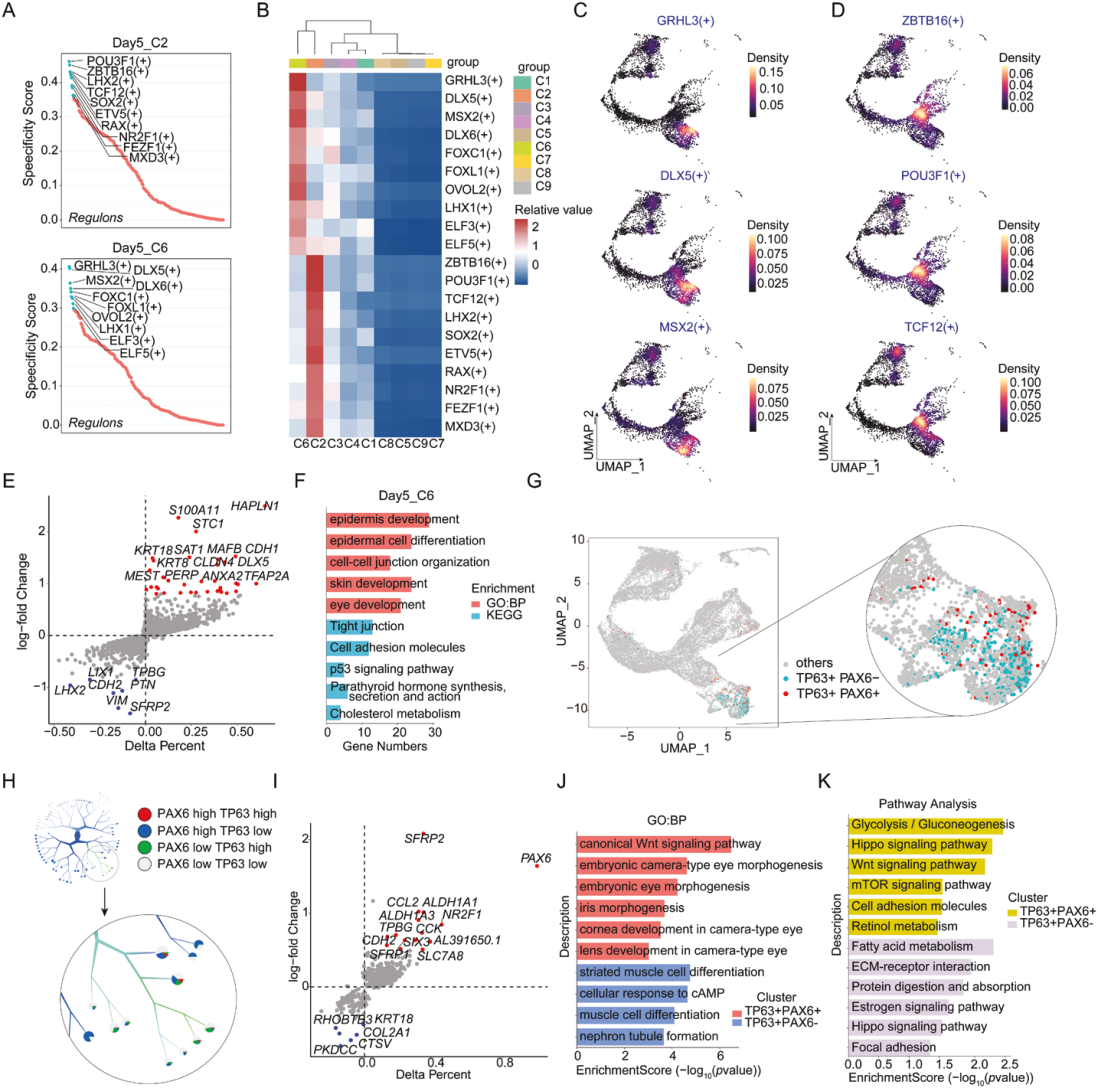
**Analysis of bidirectional neuroectoderm, surface ectoderm differentiation, and OSE differentiation.** (A) The RSS ranking plot shows key transcription factors in C2 and C6 at Day 5. (B) Heatmap of transcription factor activity scores highlighting highly active factors in subpopulations C2 and C6 at Day 5. (C) UMAP distribution of key transcription factors in subpopulation C6 at Day 5. (D) UMAP distribution of key transcription factors in subpopulation C2 at Day 5. (E) Volcano plots displaying the upregulated and downregulated differential genes between C2 and C6 at Day 5. (F) Bar plot showing top five GO and KEGG pathway enrichment terms for C6 at Day 5. (G) UMAP plot showing cell population co-expressing PAX6 and TP63. (H) PAX6 and TP63 expression tree plot using too many cells. (I) Volcano plots displaying the upregulated and downregulated differential genes between PAX6 + TP63 + and PAX6 − TP63 + populations. (J) Bar plot showing top GO enrichment terms for PAX6 + TP63 + and PAX6 − TP63 + populations. (K) Bar plot showing top KEGG pathway for PAX6 + TP63 + and PAX6 − TP63 + populations.

From the identity of cell subpopulations and the temporal UMAP distribution, we identified Day 5 as a crucial time point for cells bidirectional branching to the neuroectoderm and surface ectoderm. Therefore, we performed transcription factor analysis on subpopulations C2 and C6 of Day 5, where branching occurs. The ranking of specific scores, heatmap, and UMAP distribution results indicate that in subpopulation C2 (Fig. 5), which has the potential to differentiate into neuroectoderm, transcription factors such as ZBTB16, POU3F1, TCF12, LHX2, and RAX exhibit strong transcriptional activity. In contrast, in subpopulation C6, which differentiates into surface ectoderm, transcription factors such as GRHL3, DLX5, MSX2, and FOXC1 show strong transcriptional activity (Fig. 5A–D). These results are consistent with prior knowledge of ectodermal development, where transcription factors like POU3F1 and LHX2 are crucial for neuroectodermal pathways, and GRHL3 and FOXC1 are key regulators of surface ectodermal differentiation [42]. Importantly, our study also provides new insights into potential transcription factors involved in determining neuroectodermal and surface ectodermal fates, such as ZBTB16 and DLX5, which may have previously unrecognized roles in these processes.

The differential expressed gene analysis for C2 and C6 at Day 5 revealed distinct gene expression profiles. In C6, high expression was observed for genes such as HAPLN1, S100A11, CDH1, and KRT18 while C2 exhibited high expression of genes like SFRP2, LHX2, and VIM (Fig. 5E). GO enrichment analysis indicated that the differential genes in C6 are associated with processes such as epithelial development and differentiation, skin development, and eye development, consistent with surface ectoderm characteristics (Fig. 5F). KEGG pathway analysis suggested that pathways such as Tight junction, cell adhesion, p53, parathyroid hormone and cholesterol metabolism may be activated in C6 (Fig. 5F). Further experimental validation of these findings could potentially uncover novel regulatory networks and provide deeper insights into the developmental biology of the ectoderm.

The OSE originates from the surface ectoderm and, while maintaining surface ectoderm characteristics, also expresses the neuro-related transcription factor PAX6. Therefore, PAX6 and TP63 dual expression, localized in the surface ectoderm lineage, serve as markers for identifying ocular surface ectoderm or its precursor cells. Through UMAP expression analysis of PAX6 and TP63, we identified a PAX6 + TP63 + population distributed in C5, indicative of surface ectoderm populations (Fig. 5G). Gene expression tree analysis using too many cells also revealed a cell cluster with high expression of both PAX6 and TP63 (Fig. 5H). Furthermore, the PAX6 + TP63 + population, compared to the PAX6 − TP63 + population, exhibited higher expression of genes such as SFRP2, ALDH1A1, ALDH3A1, and SIX3, indicating similarities to corneal limbal stem cells and neuroectoderm (Fig. 5I). GO enrichment analysis showed that the PAX6 + TP63 + population was associated with embryonic eye development, corneal development, and Wnt pathway activation. In contrast, the PAX6 − TP63 + population was linked to stratified epithelium development (Fig. 5J), a typical differentiation destination of surface ectoderm. Pathway analysis further demonstrated that the PAX6 + TP63 + population exhibited higher activity in glycolysis, Hippo, Wnt, mTOR, and retinol metabolism pathways. The PAX6 − TP63 + population was more related to fatty acid metabolism, ECM–receptor interaction, and protein degradation pathways (Fig. 5K). These results indicated that we identified a population of ocular surface ectoderm cells within the OSE differentiation system and preliminarily characterized their molecular features.

### Validation of OSE differentiation system through comparative analysis with mouse embryonic development

**Figure 6.**
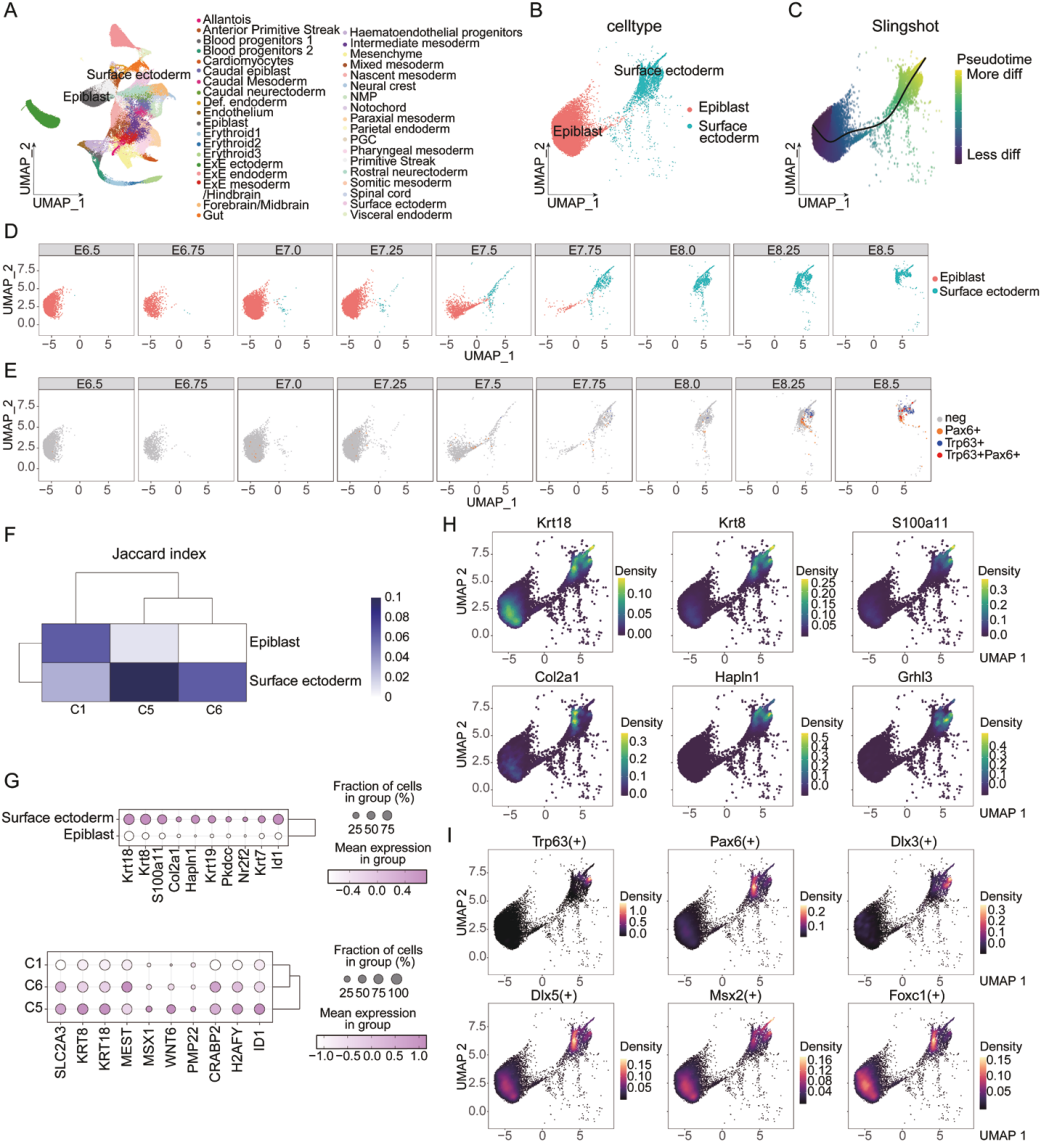
**Comparative validation of the OSE differentiation system with mouse embryonic development.** (A) UMAP plot reproducing the 37 major cell groups identified in mouse embryos from E6.5 to E8.5, including the epiblast and surface ectoderm. (B) UMAP plot showing the extracted epiblast and surface ectoderm cell groups. (C) Slingshot pseudotime analysis illustrating the differentiation pathway from the epiblast to the surface ectoderm. (D) Dynamic UMAP time facets plot showing the emergence of the surface ectoderm. (E) Dynamic UMAP time facets plot showing the emergence of the Trp63 + Pax6 + population in the surface ectoderm. (F) Comparative Jaccard index analysis showing similarity between mouse embryo and OSE system. (G) Dot plot showing the expression of surface ectoderm characteristic genes in the mouse embryo and OSE groups. (H) UMAP plots comparing the expression distribution of key surface ectoderm genes in mouse embryo data. (I) UMAP density plot of transcription factor activity analysis of key surface ectoderm transcription factors in mouse embryos.

The OSE differentiation system is an *in vitro* differentiation system, so testing the similarity of molecular mechanisms during *in vivo* development is crucial for the reliability of this system. The period from embryonic Day 6.5 (E6.5) to Day 8.5 (E8.5) is a critical stage for gastrulation and early organogenesis in mice, as well as a key phase for the fate determination of the epiblast to surface ectoderm and OSE. Blanca et al. collected 411 complete mouse embryos at six time points from E6.5 to E8.5, obtaining 116,312 single-cell transcriptomes and identifying 37 major cell groups, including the epiblast and surface ectoderm [29]. Therefore, we reanalyzed this single-cell cohort. The 37 major cell groups identified in the continuous time-point single-cell sequencing results of mouse embryos were reproduced (Fig. 6A), including the epiblast and surface ectoderm. After extracting these two groups (Fig. 6B), slingshot pseudotime analysis [28] revealed a clear differentiation pathway from the epiblast to the surface ectoderm (Fig. 6C). By observing the dynamic process of epiblast differentiation into surface ectoderm using UMAP continuous time facets, we found that the surface ectoderm appeared from E7.0 and began to boost at E7.5 (Fig. 6D). Intriguingly, from E8.25, a group characterized by Trp63 + Pax6 + emerged within the surface ectoderm (Fig. 6E), marking the ocular surface ectoderm, which is consistent with the results of our OSE differentiation system.

By calculating and comparing the Jaccard index [43, 44], we evaluated the similarity between the epiblast and surface ectoderm in mouse embryos and the corresponding groups C1, C5, and C6 in the OSE system. The results showed a higher similarity between the epiblast and C1 (ESC characteristics), and between the surface ectoderm and C5, C6 (surface ectoderm characteristics), particularly the more differentiated C5 (Fig. 6F). The expression of surface ectoderm characteristic genes in the mouse embryo groups and OSE groups was also consistently validated in each other’s datasets (Fig. 6G). The UMAP expression distribution of key surface ectoderm genes such as K*rt*8 and K*rt*18 in the OSE system was also confirmed in mouse embryo data (Fig. 6H). Further transcription factor analysis showed that key surface ectoderm transcription factors like Trp63, Dlx3, and Foxc1 had strong activity in the surface ectoderm group of mouse embryos, along with a group showing high transcriptional activity of Pax6, indicating the occurrence of ocular surface ectoderm fate determination (Fig. 6I). The similarity validation between the OSE *in vitro* differentiation and the mouse embryonic development demonstrates that the established OSE *in vitro* differentiation system faithfully recapitulates the key developmental events and molecular signatures observed during the *in vivo* ocular surface ectoderm specification process. The identification of the PAX6 + TP63 + population, representing the ocular surface ectoderm lineage, at similar developmental stages in both the *in vitro* and *in vivo* contexts further validates the reliability and relevance of this OSE differentiation model for dissecting the underlying molecular mechanisms governing ocular surface development.

### Cellular differentiation dynamics and key regulatory mechanisms atlas

**Figure 7.**
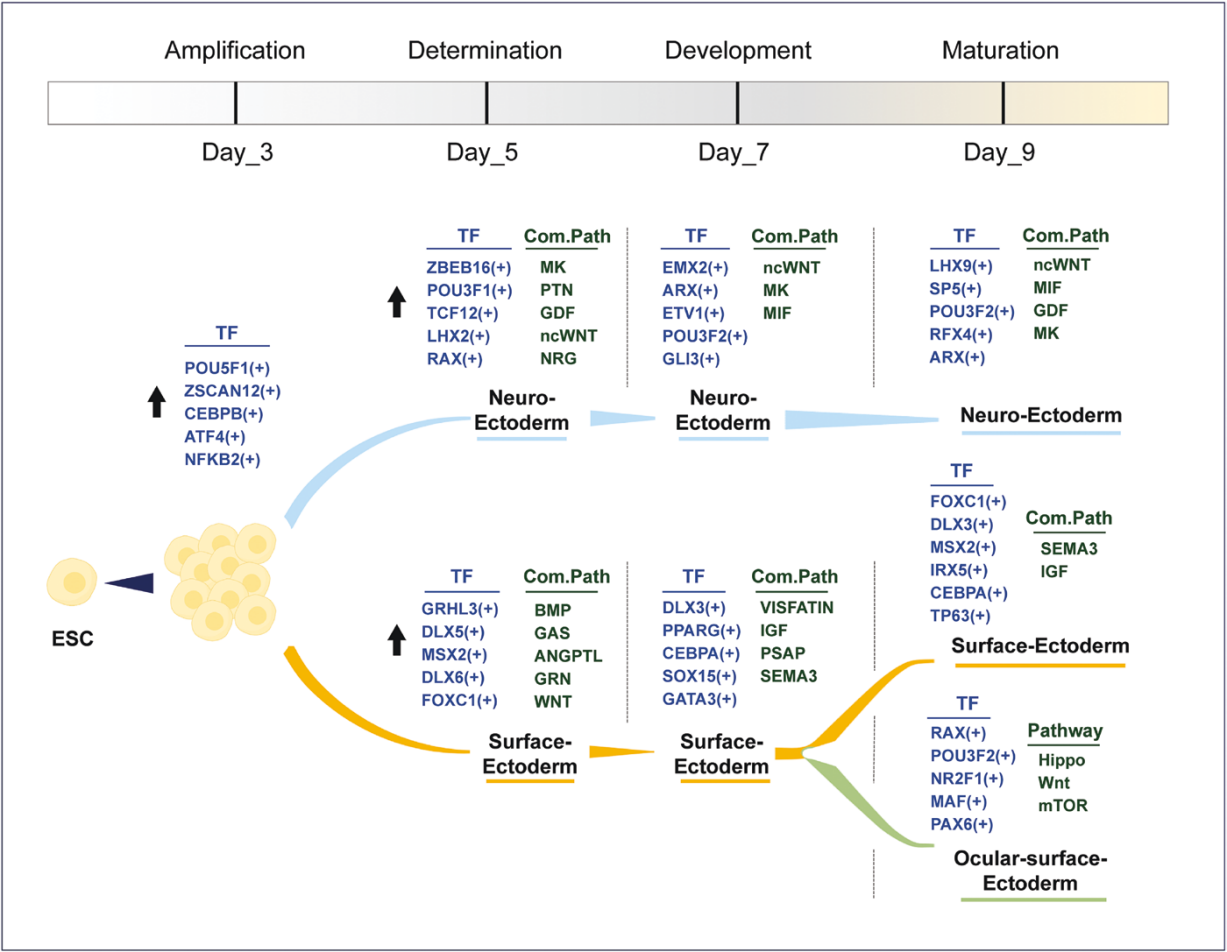
**Atlas of OSE differentiation dynamics and key regulatory mechanisms.** This schematic diagram presents the differentiation pathway from ESCs to ectodermal lineages over 9 days. ESCs initially express key transcription factors. By Day 5, cells diverge into neuro-ectodermal and surface-ectodermal lineages, influenced by specific signaling pathways. On Day 7, further specialization occurs, and by Day 9, cells mature into distinct neuro- and surface-ectodermal types, with subsets differentiating into ocular–surface–ectodermal cells. Key transcription factors and signaling pathways are marked at their respective positions in the diagram.

Based on the multi-timepoint single-cell multimodal analysis described above, we established a dynamic molecular atlas tracing the differentiation pathway from ESCs to distinct ectodermal lineages, including key transcription factors and cell–cell communication signals (Fig. 7). The cellular differentiation pathway from ESCs to distinct ectodermal lineages was monitored by single-cell RNA sequencing over a span of 9 days, delineated into four distinct stages: amplification, determination, development, and maturation (Day 3, Day 5, Day 7, and Day 9, respectively).

Initially, ESCs underwent amplification characterized by increased expression of key transcription factors such as POU5F1, ZSCAN12, CEBPB, ATF4, and NFKB2 by Day 3. By Day 5, cells began the determination phase, exhibiting divergence into neuro-ectodermal and surface-ectodermal lineages. Neuro-ectodermal determination was marked by the upregulation of transcription factors including ZEBEB16, POU3F1, TCF12, LHX2, and RAX. Concurrently, surface-ectodermal determination was indicated by enhanced expression of GRHL3, DLX5, MSX2, DLX6, and FOXC1. These lineage-specific commitments involved associated signaling pathways (Com.Path) such as MK, PTN, GDF, ncWNT, and NRG for neuro-ectoderm, and BMP, GAS, ANGPTL, GRN, and WNT for surface-ectoderm. Progressing to Day 7, during the development stage, neuro-ectodermal cells showed further specialization with transcription factors EMX2, ARX, ETV1, POU3F2, and GLI3 coming into play (Fig. S6A), alongside the continuation of ncWNT, MK, and MIF signaling pathways. Surface-ectodermal cells expressed TFs like DLX3, PPARG, CEBPA, SOX15, and GATA3 (Fig. S6A), modulated by VISFATIN, IGF, PSAP, and SEMA3 pathways. By Day 9, cells reached maturation. Neuro-ectodermal cells exhibited the presence of LHX9, SP5, POU3F2, RFX4, and ARX (Fig. S6B) TFs along with ncWNT, MIF, GDF, and MK pathways. Surface-ectodermal cells expressed TFs such as FOXC1, DLX3, MSX2, IRX5, CEBPA, and TP63 (Fig. S6B), regulated by SEMA3 and IGF pathways. A subset differentiated into ocular-surface-ectodermal cells, showing a unique expression pattern of transcription factors like RAX, POU3F2, NR2F1, MAF, and PAX6 (Fig. S6C) with Hippo, Wnt, and mTOR signaling pathways. These findings underscore the intricate temporal dynamics of transcription factor activation and pathway engagement crucial for ectodermal lineage bifurcation and maturation from ESCs.

## Discussion

The present study provides a comprehensive single-cell transcriptomic analysis of the *in vitro* OSE differentiation system, revealing dynamic cellular trajectories, key transcription factors, and signaling pathways that govern the bifurcation and maturation of ectodermal lineages. By integrating multiple analytical tools, we were able to delineate the differentiation process from ESC into distinct ectodermal fates, including the neuroectoderm, surface ectoderm, and ocular surface ectoderm.

One of the key findings is the identification of three distinct differentiation branches within the OSE system: the neural crest lineage, the neuroectodermal lineage, and the surface ectodermal lineage. The bifurcation points at Day 5 emerged as a critical juncture where cells began to commit to either the neuroectoderm or surface ectoderm lineages. This bifurcation was marked by distinct transcriptional profiles in subpopulations C2 and C6, with C2 expressing neuroectoderm-specific markers and C6 expressing surface ectoderm-specific markers. This branching pattern is consistent with the established understanding that the ocular surface ectoderm originates from the surface ectoderm, while also maintaining a close relationship with the neuroectoderm [3].

The transcription factor analysis provides important insights into the regulatory mechanisms underlying the differentiation process. We identified known transcription factors, such as FOXC1 and RXRA, which are crucial for surface ectoderm development [45, 46], as well as novel factors, such as ZNF816, that may play previously unrecognized roles in this process. Furthermore, the distinct transcription factor profiles observed in the neuroectodermal and surface ectodermal lineages, including ZBTB16, POU3F1, TCF12, LHX2, RAX, GRHL3, DLX5, MSX2, and FOXC1, highlight the intricate molecular mechanisms that govern the bifurcation of these two major ectodermal fates.

Importantly, a PAX6 + TP63 + cell population was identified within the surface ectoderm lineage, which represents the OSE or its precursor cells. This population exhibited a unique gene expression profile, including higher expression of genes associated with corneal limbal stem cells and the neuroectoderm, suggesting a distinct molecular identity. The enrichment of pathways related to embryonic eye development, corneal development, and Wnt signaling in this population further supports its identity as the ocular surface ectoderm.

Our *in vitro* model successfully recapitulated cell groups and differentiation trajectories observed in mouse embryonic development stages E6.5–E8.5. This was validated by pseudotime analysis, which showed differentiation from epiblast to surface ectoderm, along with the emergence of Trp63 + Pax6 + ocular surface ectoderm cells at E8.25, consistent with *in vivo* findings. The high Jaccard index and consistent gene expression patterns further validated the reliability of our *in vitro* system compared to *in vivo* development.

Moreover, the differentiated corneal epithelial cells from this induced system could serve as a potential cellular source for corneal tissue, offering clinical applications such as corneal transplantation. This potential application underscores the translational significance of our findings and suggests a promising avenue for regenerative medicine and therapeutic interventions for patients requiring corneal grafts.

## Research limitations

Despite revealing valuable insights into the single-cell transcriptomic dynamics of OSE differentiation, our *in vitro* model may not fully replicate the complexity of the *in vivo* environment, including cell–cell interactions and extracellular matrix influences. Additionally, the analysis covered only four time points, potentially missing critical intermediate states and transient dynamics. The depth of single-cell RNA sequencing and technical variability might also affect the identification of rare cell populations and the completeness of gene expression profiles. Lastly, while key transcription factors and signaling pathways were identified, further functional validation is necessary to confirm their roles in the differentiation process. Future studies could employ CRISPR-Cas9 mediated knockouts, overexpression studies, RNA interference, and the use of chemical inhibitors to validate the functional roles of these factors and pathways, thereby addressing these limitations.

## Methods

### Research ethics

The human ESC cell line H1 (WA-01) was kindly provided by Professor Nan Cao from the Sun Yat-Sen University, Zhongshan School of Medicine. All procedures involving ESCs were performed in accordance with the principles of the Declaration of Helsinki and the ethical guidelines provided by the International Society for Stem Cell Research.

### Cell culture and differentiation induction

ESCs were cultured on human ESC-qualified Matrigel (Corning)-coated plates using mTeSRTM1 medium (Stem Cell Technologies), as previously described [47, 48]. The differentiation of ESCs was carried out according to previous reports, with some modifications [12, 31]. Briefly, ESC cells were dissociated using GCDR (Gentle Cell Dissociation Reagent, Stem Cell Technologies) and resuspended in a 500 μL medium. After centrifugation, cell aggregates were seeded in the center of six-well plates precoated with LN511E (Laminin-511-E), and then cultured in mTeSRTM1 medium for 3–7 days. The medium was then changed to differentiation medium (DM), which consisted of GMEM (Glasgow’s Minimum Essential Medium, Life Technologies) supplemented with 10% KSR (knockout serum replacement, Life Technologies), 1 mM sodium pyruvate, 0.1 mm non-essential amino acids, 2 mM l-glutamine, 1% penicillin–streptomycin solution, and 55 μM 2-mercaptoethanol (all from Life Technologies). After 3 weeks, the cultures were observed to contain OSE, NE (Neuroectoderm), and GSE cells, which exhibited distinct morphologies and clear delineation between cell types. To achieve corneal epithelial cell differentiation, the cells were further cultured in corneal differentiation medium (CDM; DM and Cnt-PR (1:1, CELLnTEC Advanced Cell Systems)) containing 20 ng/mL KGF (Keratinocyte Growth Factor, Wako) and 10 μM Y-27632 (Wako) for 6 weeks, followed by culture in corneal epithelium culture medium (CECM; DMEM/F12 and DMEM (1:1)) supplemented with 10% fetal bovine serum (Gibco), 10 ng/mL EGF (Millipore), 5 μg/mL insulin (Sigma), 0.4 μg/mL hydrocortisone (Millipore), 0.1 nM cholera toxin (Sigma), 2 nM 3,3ʹ,5-triiodo-l-thyronine (Sigma), and 1% penicillin/streptomycin (Gibco) for an additional 2 weeks [31].

### Immunofluorescence imaging

Cell samples were examined using bright field and immunofluorescence microscopy techniques. Samples were fixed, permeabilized, and blocked using standard protocols. Cells were then incubated with primary antibodies overnight at 4°C. After washing, samples were incubated with appropriate fluorescently labeled secondary antibodies for 1 h at room temperature. Nuclei were counterstained with Hoechst 33342 (1:1000; Thermo Fisher Scientific, USA). The primary antibodies used for immunofluorescence are as follows: anti-TP63 (GeneTex, # GTX102425, 1:200), anti-PAX6 (BioLegend, #939802, anti-KRT3 (Abcam, #ab68260, 1:200), anti-KRT4 (GeneTex, #GTX112211, 1:200), anti-KRT18 (Abcam, # ab1191, 1:200), anti- KRT8 (Abcam, # ab15823, 1:200), antirabbit IgG (Alexa Fluor 488 Conjugate, CST, 4412 S,1:1000), antimouse IgG (Alexa Fluor 488 Conjugate, CST, 4408 S,1:1000), antirabbit IgG (Alexa Fluor 594 Conjugate, CST, 8889 S,1:1000), and antimouse IgG (Alexa Fluor 594 Conjugate, CST, 8890 S,1:1000). Images were captured using LSM 800 microscope (ZEISS, Germany).

### Single-cell sample preparation and sequencing

Cells from the OSE differentiation system were collected at four distinct time points: Day 3, Day 5, Day 7, and Day 9 after induction. A total of 22,328 cells were isolated for single-cell RNA sequencing. Libraries were prepared following the manufacturer’s protocol. Single-cell RNA sequencing was performed using the 10× genomics chromium platform.

### Data preprocessing and quality control

Raw sequencing data were processed using the Cell Ranger pipeline (10× Genomics) to generate a gene-barcode matrix. The Seurat R package (4.4.0) was utilized for quality control. Cells with < 200 detected genes or > 10% mitochondrial RNA were excluded to ensure data quality. Genes expressed in fewer than three cells were also filtered out. Filtered data were normalized and scaled using Seurat’s standard workflow. Feature selection was conducted to identify highly variable genes. Differential gene expression analysis was conducted using the FindMarkers function in Seurat.

### Dimensionality reduction and clustering

UMAP was applied for dimensionality reduction to visualize cell populations. Louvain clustering was performed through Seurat to identify distinct cell subgroups. This unsupervised clustering revealed nine subgroups, labeled C1–C9. Cell cycle scoring was performed using Seurat’s CellCycleScoring function to assign cells to G1, S, or G2M phases based on the expression of cell cycle-related genes.

### GO and pathway analysis

GO: BP enrichment analysis was conducted on upregulated differential genes in each cell subgroup using the clusterProfiler R package (4.10.0). Pathway enrichment analysis was conducted using the KEGG database through the clusterProfiler R package.

### Pseudotemporal inference

R package VECTOR was employed to infer vectors of developmental directions for cells within UMAP projections, identifying initial development positions and trajectories. Monocle3 (1.3.6) and Palantir (1.3.2) algorithms were used to infer pseudotemporal pathways, revealing three distinct differentiation branches within the OSE system. Additionally, the Slingshot (2.10.0) algorithm was applied to the UMAP embeddings to further define pseudotemporal trajectories in mouse embryo data.

### Cell communication and transcription factor analysis

R package Cellchat (1.6.1) was used to explore changes in ligand–receptor signaling pathways between cell subpopulations over time, providing insights into intercellular communication dynamics. The pySCENIC algorithm (0.12.1) was utilized to calculate transcription factor scores for each cell subpopulation, identifying key regulators and their activity distribution.

### Cluster correlation analysis

Pearson correlation analysis was employed to examine the relationships between gene expression profiles across different cell subpopulations. Gene expression data were normalized and log-transformed using the Seurat package. Correlation matrices and heatmaps were generated to visualize these relationships.

### Jaccard index calculation

The Jaccard index was calculated to compare the similarity between the epiblast and surface ectoderm in mouse embryos and corresponding groups in the OSE system. The Jaccard index is defined as the size of the intersection divided by the size of the union of the two sets. It is a measure of similarity between two sets, with values ranging from 0 to 1. A higher Jaccard index indicates a greater similarity between the sets. The formula for the Jaccard index is: Jaccard index = ∣*A*∪*B*∣/∣*A*∩*B*∣, where ∣*A*∩*B*∣ is the number of elements in the intersection of sets *A* and *B*, and ∣*A*∪*B*∣ is the number of elements in the union of sets *A* and *B*. This index was used to quantitatively assess the overlap between the gene expression profiles of the epiblast and surface ectoderm in mouse embryos and the corresponding cell groups identified in the OSE system.

### Statistical analysis

All statistical analyses were conducted using R (version 4.3.1), employing various methods to process and analyze data. Statistical methods embedded within the Seurat package were employed throughout the analysis: The raw gene expression counts were normalized using the NormalizeData function. The data were then log-transformed and scaled to account. Principal Component Analysis (PCA) was performed using the RunPCA function, and the statistically significant principal components were identified based on an elbow plot. FindClusters function applies a k-nearest neighbor (KNN) graph clustering approach based on the Euclidean distance in PCA space. Differential expression analysis was conducted using the FindMarkers function, which employs a Wilcoxon rank-sum test. *P*-values were adjusted for multiple comparisons using the Benjamini–Hochberg method to control the false discovery rate.

## Supplementary Material

lnae033_suppl_Supplementary_Figures_S1-S6

## Data Availability

This research generates no new code. All sequencing data, analysis and plotting code are available upon reasonable request.
