# Supplementary material for: Deciphering the dynamic single-cell transcriptional landscape in the ocular surface ectoderm differentiation system
Source: Life Med. 2024 Sep 5;3(5):lnae033. doi: 10.1093/lifemedi/lnae033 (PMC11749776; doi:10.1093/lifemedi/lnae033)
Supplement: lnae033_suppl_Supplementary_Figures_S1-S6 [file lnae033_suppl_Supplementary_Figures_S1-S6.pdf]

Supplementary Figures:

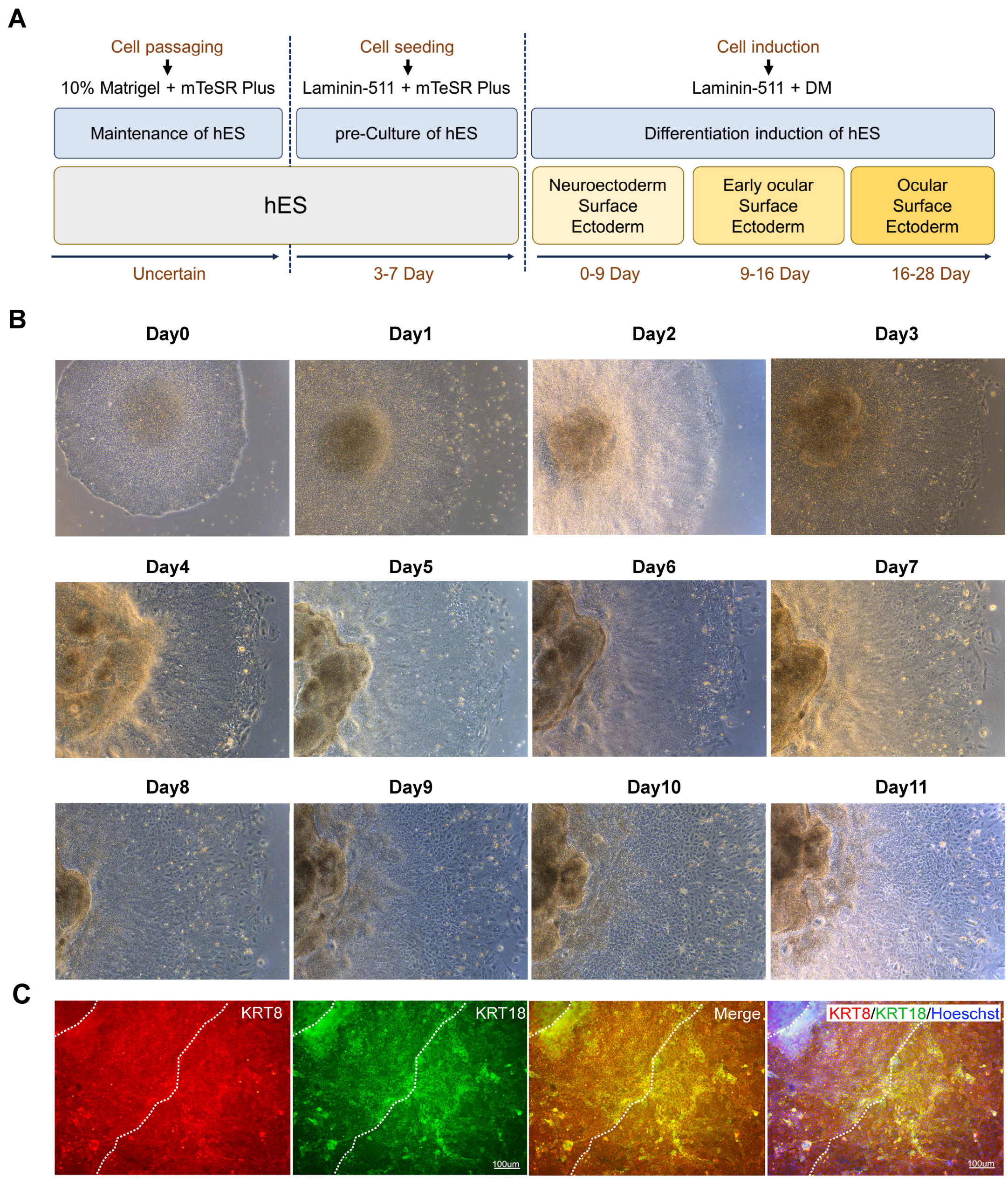

Figure S1. Methodology for OSE differentiation from ESCs and progressive phase contrast morphology.

(A) Schematic diagram outlining the protocol for inducing ocular surface ectoderm differentiation from ESCs. (B) Sequential phase-contrast microscopy images showing the morphological changes of ESCs during the early stages of differentiation from Day 0 to Day 11. Each image represents a specific day in the differentiation timeline. (C) Immunofluorescence staining for KRT8 and KRT18 of SEAM.

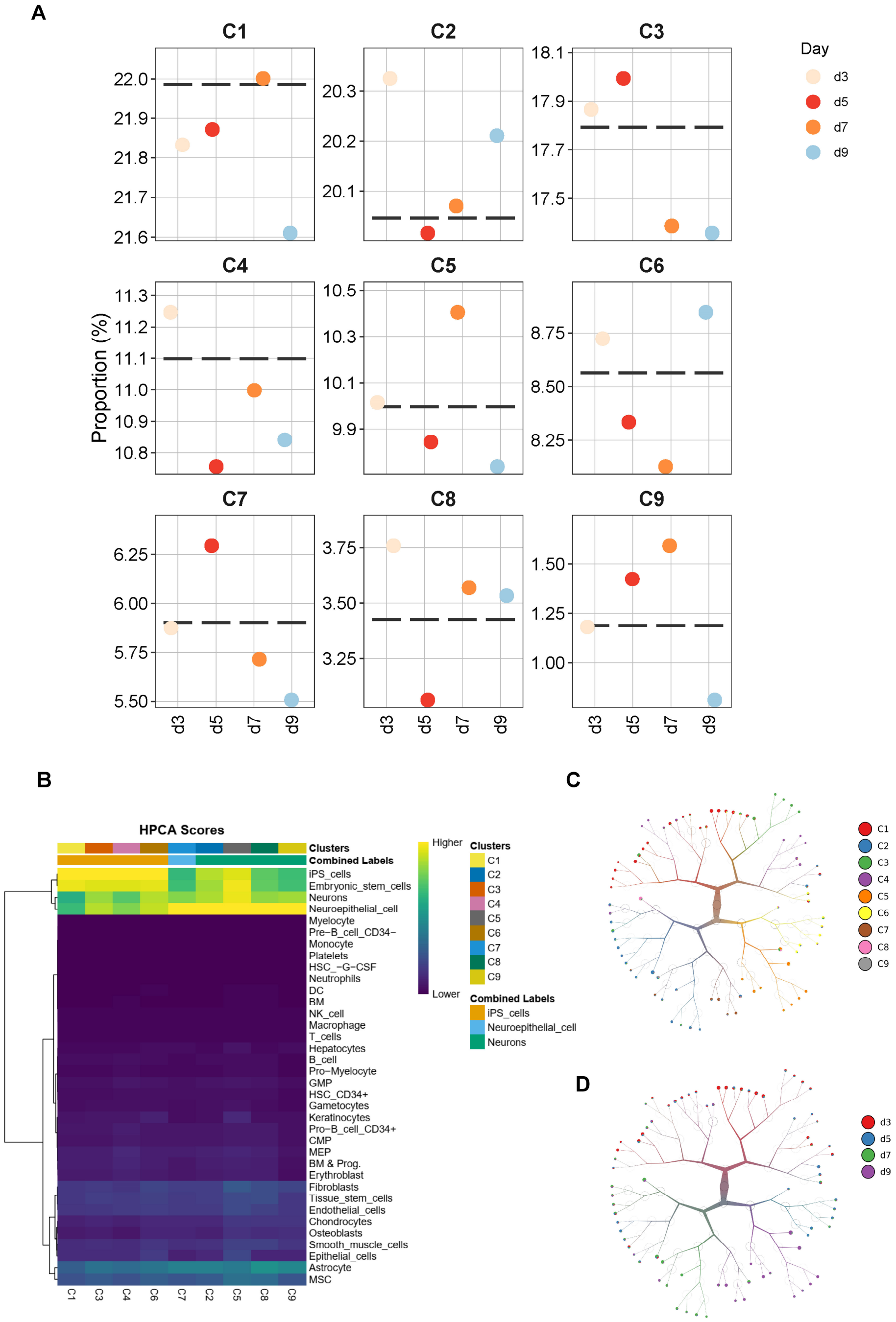

Figure S2. Temporal and subpopulation dynamics of differentiated cells in the OSE differentiation system.

(A) Dot plot showing the cell proportion by subgroups over time. (B) Heatmap showing the automatic cell annotation results from singleR. (C) toomanycells branching analysis plot illustrating the emergence and increase of differentiated cells over time. (D) Temporal distribution of cell subpopulations from the toomanycells analysis.

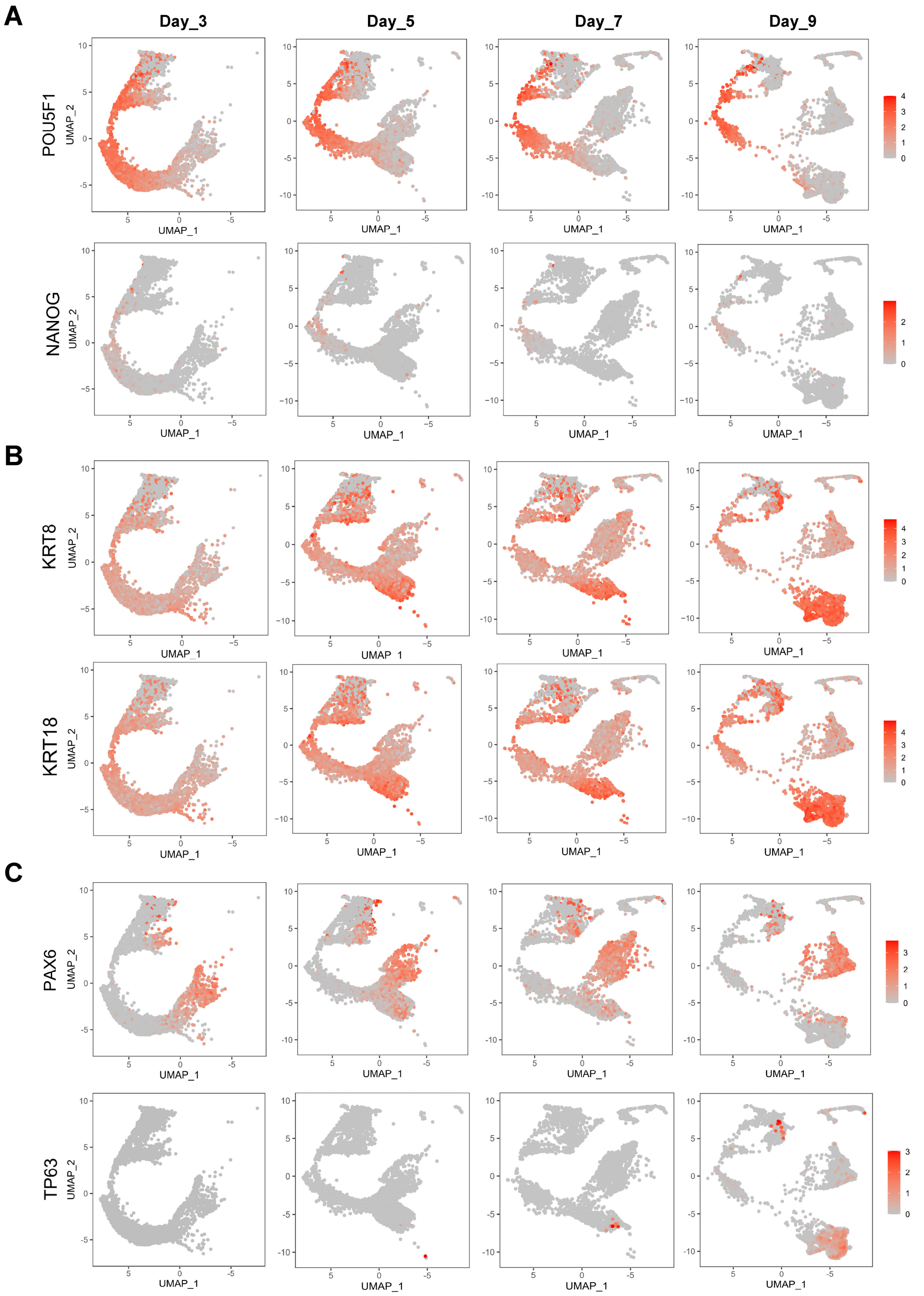

Figure S3. Time-series UMAP facet expression of differentiation markers in the OSE differentiation system.

(A) Time-series UMAP facet expression of ESC markers POU5F1 and NANOG. (B) Time-series UMAP facet expression of surface ectoderm markers KRT8 and KRT18. (C) Time-series UMAP facet expression of OSE markers PAX6 and TP63.

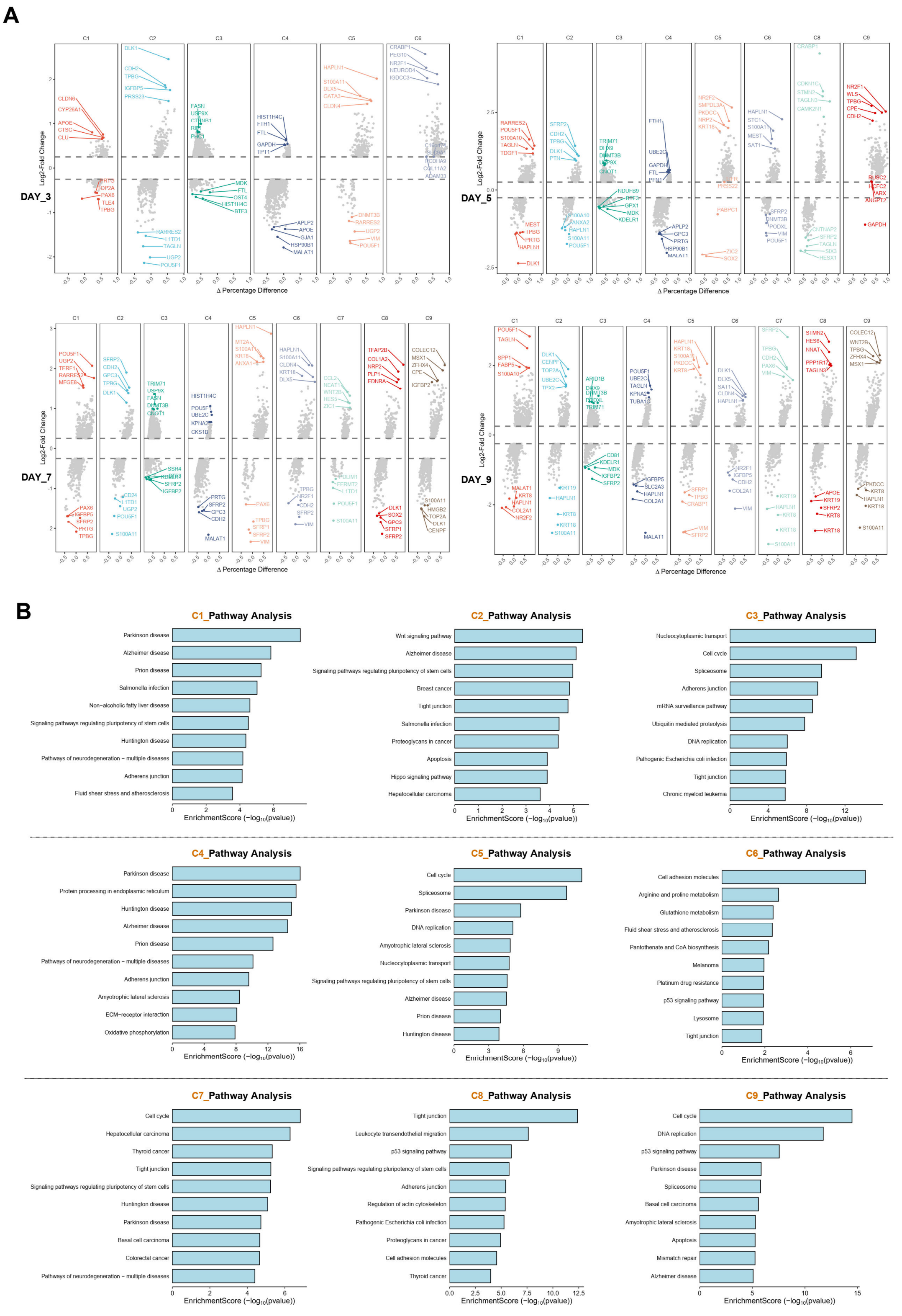

Figure S4. Volcano plots of marker genes at various time points and KEGG enrichment analysis for different cell subpopulations.

(A) Volcano plots of marker genes for each cell subpopulation at different time points. (B) Top 10 KEGG enrichment analysis results for marker genes of all

subpopulations.

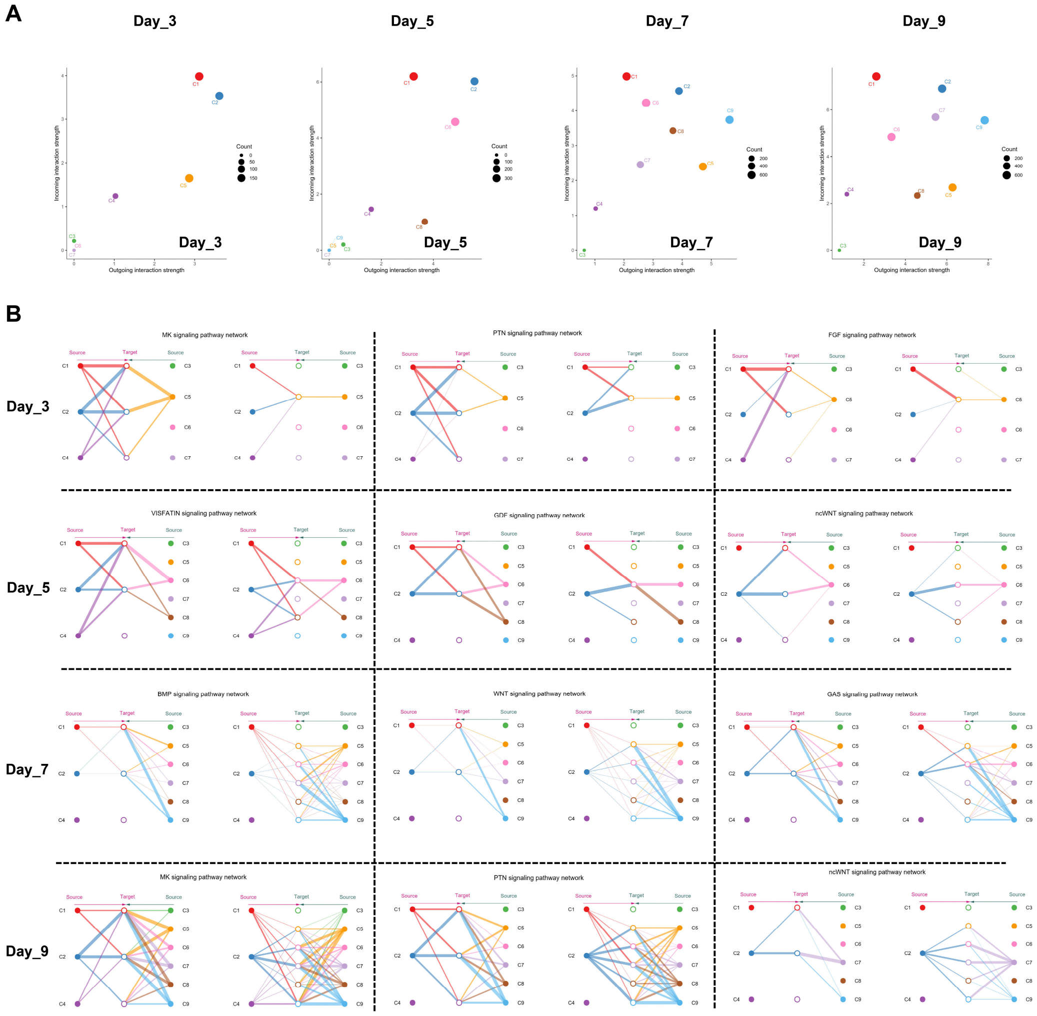

Figure S5. Dynamic changes in ligand–receptor signaling pathways and transcription factor activity over time in the OSE differentiation system.

(A) Scatterplot showing the number of active incoming and outgoing communications over time in cell subpopulations. (B) Hierarchical plot depicting detailed intercellular communication pathways active between subpopulations from day 3 to day 9.

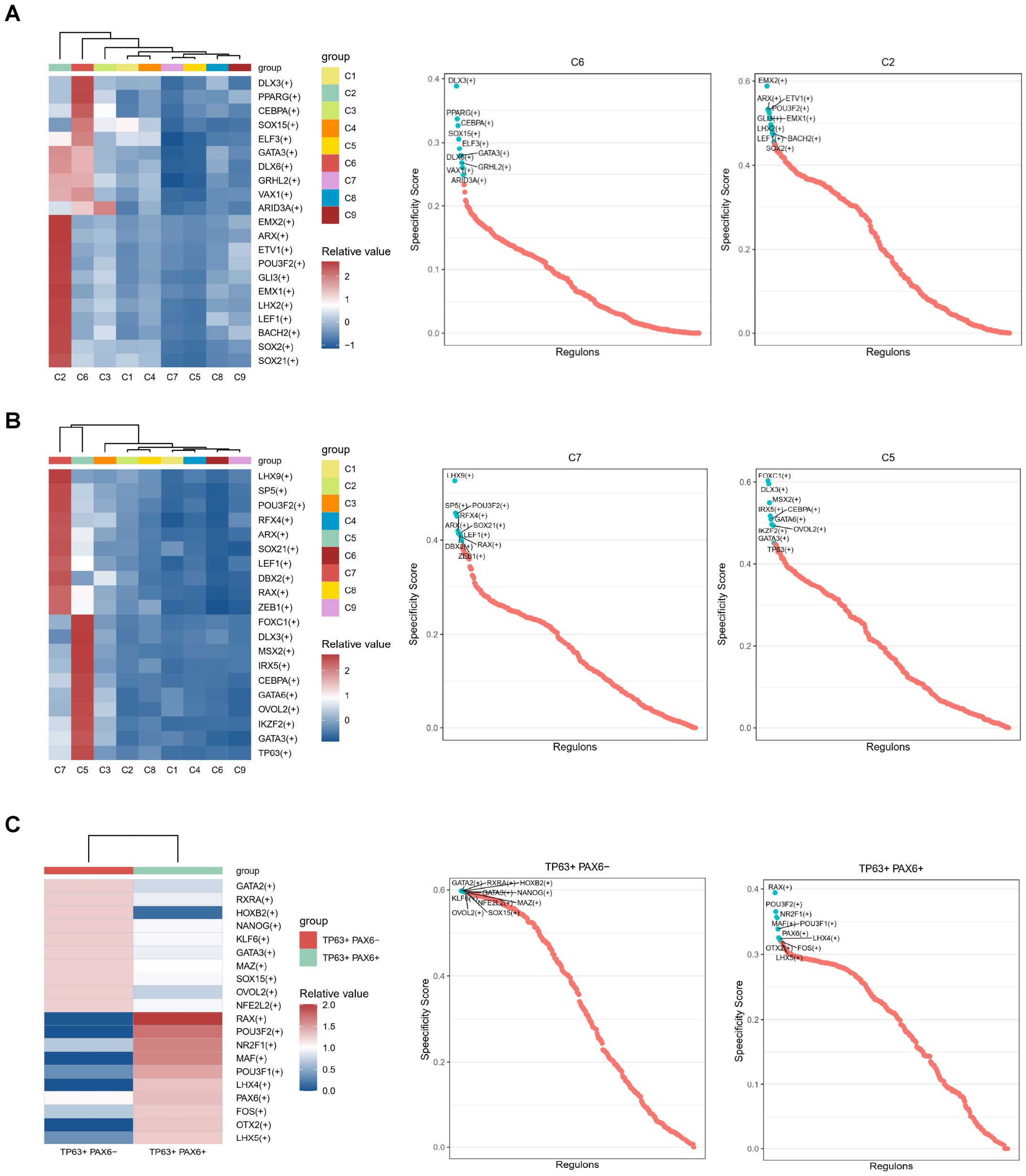

Figure S6. Transcription factors analysis across time points in neuroectoderm, surface ectoderm and OSE lineage.

(A) Heatmap (left) of transcription factor activity scores highlighting highly active factors in subpopulations C2 and C6 at day 7. Ranking (right) of the transcription factors by Specificity Score for group C6 and C2 at day 7. (B) Heatmap (left) of transcription factor activity scores highlighting highly active factors in subpopulations C7 and C5 at day 9. Ranking (right) of the transcription factors by Specificity Score for group C7 and C5 at day 9. (C) Heatmap (left) of transcription factor activity scores highlighting highly active factors in PAX6+TP63+ and PAX6−TP63+ populations.

Ranking (right) of the transcription factors by Specificity Score for PAX6+TP63+ and PAX6−TP63+ populations.
